# Dataset on LC-Q-TOF/MS tentative identification of phytochemicals in the extract of *Vernonia amygdalina* leaf through positive ionization

**DOI:** 10.1016/j.dib.2018.10.159

**Published:** 2018-11-03

**Authors:** Oluwaseun Ruth Alara, Nour Hamid Abdurahman, Chinonso Ishmael Ukaegbu, Zulkafli Hassan, Nassereldeen Ahmed Kabbashi

**Affiliations:** a Centre of Excellence for Advanced Research in Fluid Flow (CARIFF), Universiti Malaysia Pahang, Lebuhraya Tun Razak, 26300 Gambang, Pahang, Malaysia; b Faculty of Industrial Sciences and Technology, Universiti Malaysia Pahang, Lebuhraya Tun Razak, 26300 Gambang, Pahang, Malaysia; c Faculty of Chemical and Natural Resources Engineering, Universiti Malaysia Pahang, Lebuhraya Tun Razak, 26300 Gambang, Pahang, Malaysia; d Bioenvironmental Engineering Research Centre (BERC), Department of Biotechnology Engineering (BTE), Kulliyyah of Engineering (KOE), International Islamic University Malaysia, Gombak, 50728 Kuala Lumpur, Malaysia

## Abstract

The tentative identification of bioactive compounds in the extract of *Vernonia amygdalina* leaf was carried out using positive ionization of Liquid chromatography-mass spectrometry quadrupole time of flight (LC-Q-TOF/MS). The positive ionization is associated with the presence of saponins, flavonoids, alkaloids, terpenoids, and glycosides. Tentative assignments of the secondary metabolites were performed by comparing the MS fragmentation patterns with Waters® UNIFY library which allows positive identification of the compounds based on the spectral match. All the metabolites compounds were estimated and presented in a BPI (Base peak intensity) plot. These data are the unpublished supplementary materials related to “Ethanolic extraction of bioactive compounds from *V. amygdalina* leaf using response surface methodology as an optimization tool” (Alara et al., 2018).

**Specification table**

**Table:** 

Subject area	Natural product research, Medicinal chemistry
More specific subject area	Analytical chemistry
Type of data	Figure, Table
How data were acquired	Liquid chromatography-mass spectrometry quadrupole time of flight
Data format	Analysed
Experimental features	The tentative estimation of secondary metabolites in *V*. *amygdalina* leaf was examined
Data source location	Centre of Excellence for Advanced Research in Fluid Flow (CARIFF), Universiti Malaysia Pahang, Gambang, Malaysia
Data accessibility	Data have been outlined within this article and Supplementary material
Related research article	Alara OR, Abdurahman NH, Olalere OA. Ethanolic extraction of bioactive compounds from *Vernonia amygdalina* leaf using response surface methodology as an optimization tool. J. Food Meas. Charact. 2018 12:1107–1122.

**Value of the data**

• The data can be used as baseline showing the present saponins, flavonoids, terpenoids, alkaloids, and glycosides in the extract of *Vernonia amygdalina* leaf.
• The data can be used to provide information on LC-Q-TOF/MS conditions of the positive ionization secondary metabolites in *V. amygdalina* leaf.
• The data presented in this manuscript can further be isolated, purified, characterized and quantified.

## 1 Data

**Fig. 1:**
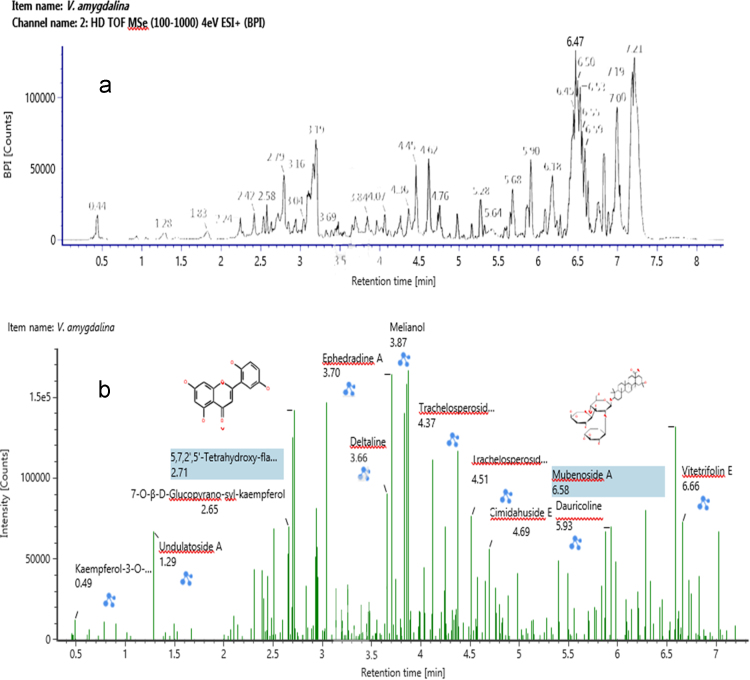
LC-Q-TOF/MS base peak intensity profiles of positive ions secondary metabolites (a) and identified compounds (b).

The data illustrate the LC-Q-TOF/MS base peak intensity profiles of saponins, flavonoids, terpenoids, alkaloids, and glycosides obtained from the extract of *V. amygdalina* leaf as compared to Waters® UNIFY library in the positive ionization (Fig. 1). The tentatively assigned secondary metabolites are presented in the Supplementary table 1.

## 2 Material, method and sample preparation

The powdered sample of *V. amygdalina* leaf collected from Gambang, Malaysia was extracted at the optimum conditions based on the previous report using an ETHOS X microwave-assisted extractor (Milestone, Italy) [1]. A 10 g sample of *V. amygdalina* leaf was irradiated for 4 min at the temperature of 70 °C, microwave power of 558 W, the solvent concentration of 76% v/v ethanol, and solid/solvent ratio of 1:10 g/mL. After the extraction process, the extract mixture was filtered using Whatman filter No. 1 and concentrated through a rotatory evaporator. Then, the extract was prepared for LC-Q-TOF/MS analysis.

The concentrated extract of *V. amygdalina* leaf was re-dissolved in HPLC grade methanol to make a concentration of 0.02 mg/mL prior to LC-Q-TOF/MS analysis. The LC-Q-TOF/MS equipped with Photodiode Array detector (Waters Vion IMS, USA) and a symmetry C_18_ column of 100 mm × 2.1 mm, 1.8 µm particle size (Waters Acquity UPLC HSS T3, USA) was employed. The mobile phases used for the analysis were A (water with 0.1% formic acid) and B (100% acetonitrile). The gradient elution as previously reported was employed [2], [3]. Briefly, the gradients elution were 90% A and 10% B (0.00 min) 90% A and 10% B (0.00–1.25 min), 45% A and 55% B (1.25–4.17 min), 10% A and 90% B (4.17–6.25 min), 90% A and 10% B (6.25–8.34 min), with the flow rate of 0.5 mL/min and injection volume of 20 µL. The metabolites in the plant extract were tentatively assigned using SYNAPT mass spectrometer (Waters) coupled with an electrospray ionization operated in positive ion mode, comprised of the following operating conditions: mass range of 100–1000 m/z, low collision energy of 4 keV, high collision energy ramp start at 10 keV, column temperature of 40 °C, sample temperature of 15 °C, gas flow of 0.5 mL/min, the source type was ESI with desolvation temperature of 550 °C, source temperature of 120 °C, cone gas of 50 L/h, capillary voltage of 1.50 kV, and desolvation gas flow rate of 800 L/h. The compounds were tentatively assigned using Waters® UNIFY Software 1.0.0.
