# Supplementary material for: Dataset on LC-Q-TOF/MS tentative identification of phytochemicals in the extract of Vernonia amygdalina leaf through positive ionization
Source: Data Brief. 2018 Nov 3;21:1686–9. doi: 10.1016/j.dib.2018.10.159 (PMC6249520; doi:10.1016/j.dib.2018.10.159)
Supplement: Supplementary file 2 — Supplementary material [file mmc2.docx]

**SUPPLEMENTARY MATERIAL**

**Table 1**

Tentatively identified bioactive compounds in the extract of *Vernonia amygdalina* leaf using positive ion mode of LC-Q-TOF/MS analysis.

| Number | Compound name | Formula | Observe neural mass (Da) | Calculated mass (Da) | Mass error (mDa) | Observed m/z (ppm) | Mass error (ppm) | Observed  RT (min) | Response (Number of ions detected by MS) | Adducts | Total fragments found |
| --- | --- | --- | --- | --- | --- | --- | --- | --- | --- | --- | --- |
| 1 | 8- Debenzoylpaeoniflorin | C_16_H_24_O_10_ | 376.1374 | 375.7374 | 0.4 | 415.1005 | 1 | 0.45 | 2919 | +K | 3 |
| 2 | Undulatoside A | C_16_H_18_O_9_ | 354.0956 | 353.5956 | 0.5 | 393.0587 | 1.3 | 0.45 | 3959 | +K | 2 |
| 3 | Germacrone | C_15_H_22_O | 218.1658 | 219.4658 | -1.3 | 241.155 | -5.3 | 0.46 | 2791 | +Na | 0 |
| 4 | Longicamphenylone | C_14_H_22_O | 206.1652 | 208.0652 | -1.9 | 229.1544 | -8.3 | 0.46 | 3807 | +Na | 0 |
| 5 | 5-Hydroxy-6,4'- dimethoxy-flavone-7-O- β-D-glucopyranoside | C_23_H_24_O_11_ | 476.1338 | 474.2338 | 1.9 | 499.123 | 3.8 | 0.47 | 2896 | +Na | 11 |
| 6 | 5,7,2',5'-Tetrahydroxy- flavone | C_15_H_10_O_6_ | 286.0473 | 286.4473 | -0.4 | 287.0546 | -1.5 | 0.49 | 11447 | +H | 1 |
| 7 | Kaempferol-3-O-β-D- glucuronide | C_21_H_18_O_12_ | 462.0799 | 461.9799 | 0.1 | 463.0872 | 0.2 | 0.49 | 12130 | +H | 7 |
| 8 | Undulatoside A | C_16_H_18_O_9_ | 354.0954 | 353.7954 | 0.3 | 377.0846 | 0.8 | 0.5 | 5733 | +Na, +H | 3 |
| 9 | Morroniside | C_17_H_26_O_11_ | 406.1479 | 405.7479 | 0.4 | 429.1371 | 0.9 | 0.61 | 3037 | +Na | 1 |
| 10 | Undulatoside A | C_16_H_18_O_9_ | 354.0954 | 353.7954 | 0.3 | 377.0846 | 0.8 | 0.63 | 6168 | +Na | 3 |
| 11 | Undulatoside A | C_16_H_18_O_9_ | 354.0946 | 354.4946 | -0.4 | 355.1019 | -1.2 | 0.72 | 2539 | +H | 0 |
| 12 | Undulatoside A | C_16_H_18_O_9_ | 354.0953 | 353.8953 | 0.2 | 377.0845 | 0.6 | 0.78 | 10988 | +Na | 0 |
| 13 | Undulatoside A | C_16_H_18_O_9_ | 354.0952 | 353.8952 | 0.2 | 377.0845 | 0.4 | 0.90 | 9833 | +Na | 1 |
| 14 | 1-O-β-D-Glucopyranosylpaeonisu O-β-1-ffrone | C_16_H_24_O_9_ | 360.1420 | 360.1420 | 0.00 | 383.1313 | 0.00 | 1.01 | 4635 | +Na | 1 |
| 15 | 7-O-Methylmorroniside | C_18_H_28_O_11_ | 420.1635 | 419.8635 | 0.3 | 443.1527 | 0.8 | 1.05 | 2322 | +Na | 1 |
| 16 | Undulatoside A | C_16_H_18_O_9_ | 354.0952 | 353.9952 | 0.1 | 377.0844 | 0.3 | 1.28 | 36550 | +Na, +H | 5 |
| 17 | Undulatoside A | C_16_H_18_O_9_ | 354.0952 | 353.9952 | 0.1 | 377.0844 | 0.3 | 1.29 | 66873 | +Na, +H | 2 |
| 18 | Aloeresin | C_19_H_22_O_9_ | 394.1247 | 395.8247 | -1.7 | 395.132 | -4.2 | 1.38 | 2062 | +H | 1 |
| 19 | Actinidioionoside | C_19_H_34_O_9_ | 406.22 | 406.52 | -0.3 | 429.2092 | -0.6 | 1.4 | 4316 | +Na | 0 |
| 20 | Cichorioside B | C_21_H_28_O_10_ | 440.1689 | 439.4689 | 0.7 | 463.1582 | 1.5 | 1.45 | 2317 | +Na | 8 |
| 21 | Bilobalide | C_15_H_18_O_8_ | 326.1003 | 325.9003 | 0.2 | 349.0896 | 0.5 | 1.49 | 9596 | +Na | 0 |
| 22 | Icariside B5_1 | C_19_H_32_O_8_ | 388.2098 | 388.1098 | 0.1 | 411.199 | 0.2 | 1.52 | 4846 | +Na | 0 |
| 23 | 7-epi-Vogeloside | C_17_H_24_O_10_ | 388.1367 | 388.3367 | -0.2 | 411.126 | -0.5 | 1.67 | 6656 | +Na | 0 |
| 24 | Actinidioionoside | C_19_H_34_O_9_ | 406.2206 | 405.9206 | 0.3 | 429.2098 | 0.7 | 2.00 | 3014 | +Na | 0 |
| 25 | Undulatoside A | C_16_H_18_O_9_ | 354.095 | 354.195 | -0.1 | 377.0842 | -0.2 | 2.04 | 2195 | +Na | 0 |
| 26 | Khellol-β-D-glucoside | C_19_H_20_O_10_ | 408.1036 | 410.1036 | -2.0 | 409.1109 | -4.9 | 2.06 | 6334 | +H | 3 |
| 27 | Cichorioside B | C_21_H_28_O_10_ | 440.1693 | 439.1693 | 1.0 | 463.1585 | 2.3 | 2.06 | 2042 | +Na | 2 |
| 28 | Ginkgolide M | C_20_H_24_O_10_ | 424.1350 | 426.035 | -1.9 | 425.1423 | -4.5 | 2.10 | 14674 | +H | 1 |
| 29 | 5-Hydroxy-4'-6-dimethoxy-flavone-7-O- β-D-glucopyranoside | C_23_H_24_O_11_ | 476.1336 | 474.3336 | 1.8 | 499.1229 | 3.6 | 2.14 | 9194 | +Na | 1 |
| 30 | prim-O-Glucosylcimifugin | C_22_H_28_O_11_ | 468.1615 | 469.8615 | -1.7 | 469.1687 | -3.6 | 2.19 | 2161 | +H | 1 |
| 31 | Roseoside_1 | C_19_H_30_O_8_ | 386.1942 | 386.0942 | 0.1 | 409.1834 | 0.3 | 2.27 | 6465 | +Na, +H | 0 |
| 32 | Icariside B5_1 | C_19_H_32_O_8_ | 388.2092 | 388.8092 | -0.6 | 411.1984 | -1.3 | 2.28 | 4059 | +Na | 1 |
| 33 | Kaempferol-3-O-β-D-glucuronide | C_21_H_18_O_12_ | 462.0800 | 461.88 | 0.2 | 463.0873 | 0.5 | 2.31 | 24829 | +H | 2 |
| 34 | 5,7-Dimethoxy-4'- hydroxyflavone-4'-O-α- L-rhamnose (1-2)-β-D-glucoside | C_29_H_34_O_14_ | 606.1946 | 605.9946 | 0.2 | 607.2019 | -0.4 | 2.31 | 2193 | +H | 3 |
| 35 | Icariside B5-1 | C_19_H_32_O_8_ | 388.2091 | 388.8091 | -0.6 | 411.1984 | -1.4 | 2.31 | 43141 | +Na | 1 |
| 36 | Icariside B5-1 | C_19_H_32_O_8_ | 388.2094 | 388.6094 | -0.4 | 411.1986 | -0.9 | 2.39 | 43091 | +Na | 2 |
| 37 | Icariside B5_1 | C_19_H_32_O_8_ | 388.2094 | 388.1094 | 0.1 | 411.1986 | -0.9 | 2.39 | 6194 | +Na | 0 |
| 38 | Roseoside_1 | C_19_H_30_O_8_ | 386.1942 | 386.5942 | -0.4 | 409.1834 | 0.2 | 2.41 | 25081 | +Na | 4 |
| 39 | Taraxacolide-1-O-β-D- glucopyranoside | C_21_H_34_O_9_ | 430.2199 | 430.6199 | -0.4 | 453.2091 | -0.9 | 2.42 | 5084 | +Na | 5 |
| 40 | Odoratin-7-O-β-D- glucoside | C_22_H_24_O_9_ | 432.1401 | 434.0401 | -1.9 | 433.1474 | -4.5 | 2.43 | 5676 | +H | 4 |
| 41 | 5,7,2',5'-Tetrahydroxy- flavone | C_15_H_10_O_6_ | 286.0474 | 286.3474 | -0.3 | 287.0547 | -1.1 | 2.44 | 39116 | +H | 0 |
| 42 | Isocolumbin | C_20_H_22_O_6_ | 358.1419 | 357.8419 | 0.3 | 359.1492 | 0.8 | 2.46 | 2069 | +H | 4 |
| 43 | Cadambine | C_27_H_32_N_2_O_10_ | 544.2062 | 543.7062 | 0.5 | 545.2135 | 1.0 | 2.46 | 2819 | +H | 12 |
| 44 | 2β-Acetoxypterodontic acid | C_17_H_24_O_4_ | 292.1679 | 291.6679 | 0.5 | 331.1311 | 1.4 | 2.47 | 2084 | +K | 2 |
| 45 | Turpinionosides E | C_19_H_32_O_8_ | 388.2088 | 389.1088 | -0.9 | 411.1980 | -2.2 | 2.48 | 13601 | +Na | 3 |
| 46 | Odoratin-7-O-β-D- glucoside | C_22_H_24_O_9_ | 432.1409 | 433.3409 | -1.2 | 433.1481 | -2.7 | 2.50 | 3034 | +H | 4 |
| 47 | Kaempferol-3-O-β-D- glucuronide | C_21_H_18_O_12_ | 462.0800 | 461.88 | 0.2 | 463.0873 | 0.3 | 2.50 | 68707 | +H | 2 |
| 48 | 5,7,2',5'-Tetrahydroxy- flavone | C_15_H_10_O_6_ | 286.0473 | 286.4473 | -0.4 | 287.0546 | -1.4 | 2.50 | 45491 | +H | 1 |
| 49 | Nelumboroside A | C_27_H_30_O_16_ | 610.1529 | 610.6529 | -0.5 | 611.1602 | -0.8 | 2.52 | 3376 | +H | 7 |
| 50 | Turpinionosides E | C_19_H_32_O_8_ | 388.2100 | 387.91 | 0.3 | 411.1993 | 0.8 | 2.57 | 4675 | +Na | 3 |
| 51 | Brazilide A | C_16_H_14_O_7_ | 318.0734 | 318.5734 | -0.5 | 319.0807 | -1.6 | 2.59 | 5166 | +H | 1 |
| 52 | Kaempferol-3-O-β-D- glucuronide | C_21_H_18_O_12_ | 462.0801 | 461.8801 | 0.2 | 463.0873 | 0.5 | 2.60 | 11928 | +H | 3 |
| 53 | Safflor yellow A | C_27_H_30_O_15_ | 594.1590 | 593.659 | 0.5 | 595.1663 | 0.9 | 2.65 | 52968 | +H, +Na | 10 |
| 54 | Chrysanthemin | C_21_H_20_O_11_ | 448.1009 | 447.7009 | 0.4 | 449.1082 | 0.8 | 2.65 | 30050 | +H | 6 |
| 55 | 7-O-β-D-Glucopyranosyl-  kaempferol | C_21_H_20_O_11_ | 448.1009 | 447.7009 | 0.4 | 449.1082 | 0.8 | 2.65 | 69734 | +H | 5 |
| 56 | 3'-Hydroxypuerarin | C_21_H_20_O_11_ | 388.2097 | 387.9097 | 0.3 | 449.1081 | 0.7 | 2.66 | 12688 | +H | 1 |
| 57 | Turpinionosides E | C_19_H_32_O_8_ | 462.1517 | 462.1517 | 0.0 | 411.1989 | -0.1 | 2.67 | 7324 | +Na | 1 |
| 58 | Lactiflorin | C_23_H_26_O_10_ | 462.1517 | 462.9517 | -0.8 | 463.1590 | -1.8 | 2.67 | 3523 | +H | 1 |
| 59 | Kaempferol-3-O-β-Dglucuronide | C_21_H_18_O_12_ | 462.0803 | 461.5803 | 0.5 | 463.0876 | 1.0 | 2.69 | 19744 | +H | 1 |
| 60 | Kaempferol-3-O-β-Dglucuronide | C_21_H_18_O_12_ | 462.0803 | 461.5803 | 0.5 | 463.0876 | 1.0 | 2.70 | 22220 | +H | 4 |
| 61 | Kaempferol-3-O-β-D- glucuronide | C_21_H_18_O_12_ | 462.0803 | 461.5803 | 0.5 | 463.0876 | 1.0 | 2.70 | 125190 | +H | 5 |
| 62 | 5,7,2',5'-Tetrahydroxy- flavone | C_15_H_10_O_6_ | 286.0477 | 286.0477 | 0.0 | 287.0550 | 0.0 | 2.71 | 142141 | +H | 2 |
| 63 | 5,7,2',5'-Tetrahydroxy- flavone | C_15_H_10_O_6_ | 286.0477 | 286.0477 | 0.0 | 287.0550 | -0.1 | 2.71 | 48304 | +H | 0 |
| 64 | Physalin E | C_28_H_32_O_11_ | 544.1953 | 543.2953 | 0.9 | 545.2026 | 1.6 | 2.72 | 6543 | +H | 8 |
| 65 | 3β- Hydrosantamarine-1-O- β-D-glucopyranoside | C_21_H_32_O_9_ | 428.2045 | 428.3045 | -0.1 | 429.2118 | -0.3 | 2.74 | 2186 | +H | 7 |
| 66 | Odoratin-7-O-β-D- glucoside | C_22_H_24_O_9_ | 432.1413 | 432.8413 | -0.7 | 433.1486 | -1.6 | 2.75 | 4153 | +H | 8 |
| 67 | Chloranoside A | C_21_H_28_O_9_ | 424.1747 | 422.7747 | 1.4 | 447.1640 | 3.1 | 2.75 | 2986 | +Na | 9 |
| 68 | Oleuropein | C_25_H_32_O_13_ | 540.1842 | 540.2842 | -0.1 | 563.1734 | -0.1 | 2.75 | 2052 | +Na | 17 |
| 69 | Luteolin-7-O- [β-D- apiofuranosyl(1-6)]β-  D-glucopyranoside | C_26_H_28_O_15_ | 580.1445 | 578.4445 | 1.7 | 603.1337 | 2.8 | 2.80 | 2909 | +Na | 3 |
| 70 | 7-O-α-L- Rhamnopyranosyl- kaempferol | C_21_H_20_O_10_ | 432.1057 | 432.1057 | 0.0 | 433.1129 | 0.0 | 2.84 | 16108 | +H | 7 |
| 71 | Yuankanin | C_27_H_30_O_14_ | 578.1634 | 578.3634 | -0.2 | 579.1707 | -0.3 | 2.84 | 32988 | +H, +Na | 17 |
| 72 | Baicalin | C_21_H_18_O_11_ | 446.0851 | 445.8851 | 0.2 | 447.0923 | 0.3 | 2.85 | 7714 | +H | 2 |
| 73 | Turpinionosides E | C_19_H_32_O_8_ | 388.2093 | 388.6093 | -0.4 | 411.1985 | -1.1 | 2.86 | 3162 | +Na | 6 |
| 74 | Bruceine G | C_20_H_26_O_8_ | 394.1645 | 392.4645 | 1.7 | 417.1537 | 4.2 | 2.89 | 6714 | +Na | 7 |
| 75 | Physalin E | C_28_H_32_O_11_ | 544.1942 | 544.4942 | -0.3 | 545.2015 | -0.5 | 2.91 | 8704 | +H | 20 |
| 76 | 7-O-α-L- Rhamnopyranosyl- kaempferol | C_21_H_20_O_10_ | 432.1056 | 432.2056 | -0.1 | 433.1129 | -0.2 | 2.93 | 51330 | +H | 5 |
| 77 | 6'-O-Benzoyl-4''- hydroxy-3''- methoxypaeoniflorin | C_31_H_34_O_14_ | 630.1935 | 631.5935 | -1.4 | 631.2007 | -2.2 | 2.93 | 3303 | +H | 18 |
| 78 | 5,7,2',5'-Tetrahydroxy- flavone | C_15_H_10_O_6_ | 286.0478 | 285.9478 | 0.1 | 287.0551 | 0.2 | 2.93 | 3857 | +H | 2 |
| 79 | Baicalin | C_21_H_18_O_11_ | 446.0851 | 445.8851 | 0.2 | 447.0924 | 0.4 | 2.94 | 14305 | +H | 2 |
| 80 | Baicalin | C_21_H_18_O_11_ | 446.0851 | 445.8851 | 0.2 | 447.0924 | 0.4 | 2.94 | 81027 | +H | 5 |
| 81 | 6-O-β-D- Glucopyranosyllactinolide1 | C_16_H_26_O_9_ | 362.1575 | 362.2575 | -0.1 | 401.1207 | -0.4 | 2.95 | 57319 | +K, +Na | 8 |
| 82 | ApocynosideⅠ flavone | C_19_H_30_O_8_ | 386.1940 | 386.294 | -0.1 | 409.1832 | -0.2 | 2.95 | 38381 | +Na | 6 |
| 83 | Turpinionosides D | C_19_H_34_O_8_ | 390.2255 | 390.0255 | 0.2 | 413.2147 | 0.4 | 2.95 | 2548 | +Na | 5 |
| 84 | Deltaline | C_27_H_41_NO_8_ | 507.2833 | 507.1833 | 0.1 | 508.2906 | 0.2 | 2.96 | 5818 | +H | 4 |
| 85 | Kaempferol 3-O-β-D- glucuronopyranosyl methyl ester | C_22_H_20_O_12_ | 476.0960 | 475.596 | 0.5 | 477.1033 | 1.1 | 3.00 | 5783 | +H | 3 |
| 86 | Picrasinoside G | C_28_H_44_O_12_ | 572.2818 | 573.7818 | -1.5 | 573.2891 | -2.6 | 3.02 | 4987 | +H | 15 |
| 87 | Blumenol C glucoside | C_19_H_32_O_7_ | 372.2150 | 372.015 | 0.2 | 395.2043 | 0.6 | 3.03 | 6439 | +Na | 1 |
| 88 | Chloranoside B | C_21_H_28_O_9_ | 424.1734 | 424.1734 | 0.0 | 447.1626 | 0.1 | 3.04 | 146939 | +Na, +K | 24 |
| 89 | Chloranoside B | C_21_H_28_O_9_ | 424.1734 | 424.1734 | 0.0 | 447.1626 | 0.1 | 3.04 | 53821 | +Na, +K | 27 |
| 90 | Cucurbitacin B | C_32_H_46_O_8_ | 558.3196 | 558.0196 | 0.3 | 559.3269 | 0.6 | 3.10 | 2969 | +H | 7 |
| 91 | Ent- eudesmane-2α,4β,11- triol 11-O-β-D-glucopyranoside | C_21_H_38_O_8_ | 418.2565 | 418.4565 | -0.2 | 441.2457 | -0.4 | 3.13 | 31348 | +Na | 6 |
| 92 | Guaianolide ixerin D | C_15_H_12_O_3_ | 240.0784 | 240.2784 | -0.2 | 241.0857 | -0.9 | 3.14 | 11439 | +H | 8 |
| 93 | Sulfoorientalol C | C_15_H_24_O_4_S | 300.1385 | 301.2385 | -1.1 | 323.1277 | -3.3 | 3.19 | 2502 | +Na | 7 |
| 94 | Chloranoside A | C_21_H_28_O_9_ | 424.1740 | 423.474 | 0.7 | 447.1632 | 1.5 | 3.19 | 18041 | +Na | 19 |
| 95 | Guaianolide ixerin D | C_15_H_12_O_3_ | 240.0791 | 239.6791 | 0.4 | 241.0864 | 1.8 | 3.20 | 11741 | +H | 12 |
| 96 | Odoratin-7-O-β-D- glucoside | C_22_H_24_O_9_ | 432.1424 | 431.8424 | 0.3 | 455.1316 | 0.8 | 3.21 | 2096 | +Na | 9 |
| 97 | Magnocurarine | C_19_H_24_NO_3_ | 314.1757 | 314.0757 | 0.1 | 353.1389 | 0.2 | 3.22 | 2613 | +K | 4 |
| 98 | ent-16α,17-Hydroxy-19- kauranoic acid | C_20_H_32_O_3_ | 320.2338 | 321.6338 | -1.4 | 343.2230 | -4.0 | 3.23 | 2614 | +Na | 3 |
| 99 | 3α-Methoxy-2,3,25,27- tetrahydro-4,7- didehydro-7- deoxyneophysalin A | C_29_H_34_O_10_ | 542.2146 | 541.6146 | 0.6 | 565.2038 | -1.1 | 3.24 | 2664 | +Na | 17 |
| 100 | Pseudolaric acid A O-β- D-glucopyranoside | C_28_H_38_O_11_ | 550.2412 | 550.4412 | -0.2 | 573.2304 | -0.4 | 3.25 | 17633 | +Na | 31 |
| 101 | Ganoderenic acid F | C_30_H_38_O_7_ | 510.2613 | 510.6613 | -0.4 | 533.2505 | -0.8 | 3.26 | 6607 | +Na | 9 |
| 102 | Odoratin-7-O-β-D- glucoside | C_22_H_24_O_9_ | 432.1424 | 431.7424 | 0.4 | 455.1316 | 0.9 | 3.26 | 33961 | +Na, +H | 14 |
| 103 | Odoratin-7-O-β-D- glucoside | C_22_H_24_O_9_ | 432.1424 | 431.7424 | 0.4 | 455.1317 | 0.9 | 3.26 | 2016 | +Na, +H | 11 |
| 104 | 6'- Acetylneoandrographoli de | C_28_H_42_O_9_ | 522.2815 | 523.5815 | -1.3 | 523.2888 | -2.6 | 3.26 | 5354 | +H | 27 |
| 105 | Ent- eudesmane-2α,4β,11-  triol 11-O-β-D- glucopyranoside | C_21_H_38_O_8_ | 418.2576 | 417.3576 | 0.9 | 441.2468 | 2.1 | 3.30 | 3757 | +Na | 11 |
| 106 | 1α,2α,3β,19α,23- Pentadroxyurs-12- en-28-oic acid-28-O-β-  D-xylopyranoside | C_35_H_56_O_11_ | 652.3836 | 651.0836 | 1.3 | 653.3909 | 2.0 | 3.32 | 2897 | +H | 65 |
| 107 | Cimicifugoside H2 | C_35_H_54_O_10_ | 634.3714 | 634.6714 | -0.3 | 635.3786 | -0.5 | 3.32 | 8766 | +H | 68 |
| 108 | Nomilinic acid | C_28_H_36_O_10_ | 532.2309 | 532.1309 | 0.1 | 533.2382 | 0.2 | 3.34 | 2069 | +H | 31 |
| 109 | 5-Hydroxy-6,4'- dimethoxy-flavone-7-O- β-D-glucopyranoside | C_23_H_24_O_11_ | 476.1325 | 475.5325 | 0.6 | 499.1217 | 1.3 | 3.36 | 2115 | +Na | 7 |
| 110 | Hesperidin-7-O- glucoside | C_22_H_24_O_11_ | 464.1312 | 464.8312 | -0.7 | 465.1385 | -1.5 | 3.37 | 2077 | +H | 7 |
| 111 | Sulfoorientalol C | C_15_H_24_O_4_S | 300.1379 | 301.7379 | -1.6 | 323.1272 | -4.9 | 3.38 | 3246 | +Na | 2 |
| 112 | 5,7,2',5'-Tetrahydroxy- flavone | C_15_H_10_O_6_ | 286.0478 | 285.9478 | 0.1 | 287.0551 | 0.4 | 3.39 | 21248 | +H | 0 |
| 113 | Cimicifugoside H2 | C_35_H_54_O_10_ | 634.3714 | 634.6714 | -0.3 | 635.3787 | -0.4 | 3.41 | 4128 | +H | 32 |
| 114 | Bruceene | C_20_H_26_O_8_ | 394.1640 | 392.964 | 1.2 | 417.1532 | 3.0 | 3.44 | 9678 | +Na | 15 |
| 115 | Guaianolide ixerin D | C15H12O3 | 240.0790 | 239.779 | 0.3 | 241.0863 | 1.4 | 3.45 | 2369 | +H | 3 |
| 116 | 6'- Acetylneoandrographoli de | C_28_H_42_O_9_ | 522.2821 | 523.0821 | -0.8 | 523.2894 | -1.5 | 3.45 | 2218 | +H | 26 |
| 117 | Yadanzioside C | C_34_H_46_O_17_ | 726.2750 | 724.775 | 1.5 | 765.2382 | 2.0 | 3.47 | 17324 | +K | 28 |
| 118 | Neoline_1 | C_24_H_39_NO_6_ | 437.2783 | 436.7783 | 0.5 | 438.2855 | 1.2 | 3.47 | 22883 | +H | 20 |
| 119 | Senbusine B | C_23_H_37_NO_6_ | 423.2628 | 422.5628 | 0.7 | 424.2701 | 1.6 | 3.48 | 17786 | +H | 16 |
| 120 | Guaianolide ixerin D | C_15_H_12_O_3_ | 240.0788 | 239.8788 | 0.2 | 241.0861 | 0.7 | 3.51 | 2410 | +H | 3 |
| 121 | Borneol 2-O-β-D- apiosyl-(1-6)-β-D-  glucoside | C_21_H_36_O_10_ | 448.2313 | 447.8313 | 0.4 | 471.2205 | 0.9 | 3.54 | 4016 | +Na | 23 |
| 122 | Magnocurarine | C_19_H_24_NO_3_ | 314.1763 | 313.5763 | 0.6 | 337.1655 | 1.9 | 3.54 | 3630 | +Na | 6 |
| 123 | Deltaline | C_27_H_41_NO_8_ | 507.2842 | 506.2842 | 1.0 | 508.2915 | 1.9 | 3.55 | 5756 | +H | 12 |
| 124 | Cimicifugoside H2 | C_35_H_54_O_10_ | 634.3720 | 634.072 | 0.3 | 635.3793 | 0.5 | 3.55 | 11052 | +H | 78 |
| 125 | Cimicifugoside H1 | C_35_H_52_O_9_ | 616.3611 | 616.3611 | 0.0 | 617.3684 | -0.1 | 3.56 | 11934 | +H | 35 |
| 126 | Chloranoside B | C_21_H_28_O_9_ | 424.1742 | 423.2742 | 0.9 | 447.1634 | 1.9 | 3.56 | 5380 | +Na | 12 |
| 127 | Scutellone H | C_29_H_38_O_7_ | 498.2619 | 498.1619 | 0.1 | 499.2692 | 0.3 | 3.59 | 3917 | +H | 7 |
| 128 | Evodionol | C_14_H_16_O_4_ | 248.1040 | 249.004 | -0.9 | 249.1113 | -3.4 | 3.60 | 2809 | +H | 0 |
| 129 | Lucidenic acid P | C_29_H_42_O_8_ | 518.2893 | 516.8893 | 1.4 | 519.2966 | 2.6 | 3.60 | 2120 | +H | 21 |
| 130 | Turpinionosides D | C_19_H_34_O_8_ | 390.2254 | 390.2254 | 0.0 | 413.2146 | 0.1 | 3.63 | 9743 | +Na | 5 |
| 131 | Esculentoside O | C_35_H_54_O_10_ | 634.3719 | 634.1719 | 0.2 | 635.3792 | 0.3 | 3.65 | 17750 | +H | 65 |
| 132 | Yadanzioside C | C_34_H_46_O_17_ | 726.2731 | 726.6731 | -0.4 | 765.2362 | -0.5 | 3.65 | 9213 | +K | 21 |
| 133 | Lucidenic acid P | C_29_H_42_O_8_ | 518.2878 | 518.3878 | -0.1 | 519.2951 | -0.2 | 3.66 | 2177 | +H | 33 |
| 134 | Deltaline | C_27_H_41_NO_8_ | 507.2831 | 507.4831 | -0.2 | 508.2903 | -0.3 | 3.66 | 90341 | +H | 11 |
| 135 | Deltaline | C_27_H_41_NO_8_ | 507.2831 | 507.4831 | -0.2 | 508.2903 | -0.3 | 3.66 | 3366 | +H | 9 |
| 136 | Ephedradine A | C_28_H_36_N_4_O_4_ | 492.2750 | 490.875 | 1.4 | 515.2643 | 2.7 | 3.69 | 4022 | +Na | 16 |
| 137 | Mudanpioside D | C_24_H_30_O_12_ | 510.1747 | 509.1747 | 1.0 | 533.1639 | 1.9 | 3.69 | 3326 | +Na | 10 |
| 138 | Ephedradine A | C_28_H_36_N_4_O_4_ | 492.2750 | 490.875 | 1.4 | 515.2643 | 2.7 | 3.70 | 164128 | +Na | 114 |
| 139 | Ephedradine A | C_28_H_36_N_4_O_4_ | 492.2727 | 493.2727 | -1.0 | 515.2619 | -1.8 | 3.70 | 8801 | +Na | 105 |
| 140 | Cimifoetiside | C_43_H_68_O_15_ | 824.4572 | 823.0572 | 1.4 | 847.4465 | 1.7 | 3.72 | 9098 | +Na | 52 |
| 141 | Scutellone H | C_29_H_38_O_7_ | 498.2628 | 497.2628 | 1.0 | 499.2701 | 2.1 | 3.72 | 2840 | +H | 7 |
| 142 | Cimidahuside E | C_35_H_52_O_8_ | 600.3664 | 600.1664 | 0.2 | 601.3737 | 0.3 | 3.75 | 37275 | +H | 69 |
| 143 | Cimidahuside H | C_35_H_54_O_9_ | 618.3768 | 618.3768 | 0.0 | 619.3841 | 0.0 | 3.76 | 2921 | +H | 45 |
| 144 | Pterodontoside H | C_21_H_36_O_7_ | 400.2460 | 400.346 | -0.1 | 423.2353 | -0.2 | 3.79 | 12882 | +Na | 6 |
| 145 | Cimicifugoside H2 | C_35_H_54_O_10_ | 634.3728 | 633.2728 | 1.1 | 635.3801 | 1.7 | 3.79 | 2402 | +H | 87 |
| 146 | Scutellone H | C_29_H_38_O_7_ | 498.2617 | 498.3617 | -0.1 | 499.2689 | -0.2 | 3.80 | 7082 | +H | 14 |
| 147 | 3-Hydroxy-12,14-  diene-27-nordeane-28- nic acid | C_29_H_42_O_4_ | 454.3085 | 454.1085 | 0.2 | 455.3158 | 0.4 | 3.80 | 2352 | +H | 28 |
| 148 | Cimidahuside H | C_35_H_54_O_9_ | 618.3774 | 617.7774 | 0.6 | 619.3847 | 1.0 | 3.83 | 6318 | +H | 74 |
| 149 | 24-O-Acetyl-7,8- didehydrocimigenol-3- O-β-D-xylopyranoside | C_37_H_58_O_11_ | 678.3990 | 677.399 | 1.0 | 701.3882 | 1.5 | 3.83 | 140253 | +Na, +H | 54 |
| 150 | Picrasidine F | C_29_H_26_N_4_O_3_ | 478.2004 | 478.3004 | -0.1 | 479.2076 | -0.3 | 3.85 | 4336 | +H | 3 |
| 151 | Melianol | C_35_H_48_O_9_ | 612.3299 | 612.2299 | 0.1 | 613.3372 | 0.2 | 3.86 | 158262 | +H | 30 |
| 152 | 1,6-O,O-Diacetyl- britannilactone | C_19_H_26_O_6_ | 350.1727 | 350.4727 | -0.3 | 373.1619 | -0.7 | 3.87 | 3172 | +Na | 1 |
| 153 | Melianol | C_35_H_48_O_9_ | 612.3300 | 612.23 | 0.1 | 613.3372 | 0.2 | 3.87 | 166864 | +H | 66 |
| 154 | Phellochinin A | C_26_H_30_O_11_ | 518.1780 | 518.978 | -0.8 | 541.1673 | -1.4 | 3.87 | 2390 | +Na | 5 |
| 155 | 3-O-β-D-  Galactopyranosyl-(1-2)-β-D-6-O- methylglucuronopyrano syl quillaic acid | C_43_H_66_O_16_ | 838.4353 | 838.2353 | 0.2 | 861.4245 | 0.2 | 3.88 | 11864 | +Na,  +H | 120 |
| 156 | Pterodontoside A | C_21_H_32_O_8_ | 412.2096 | 412.3096 | -0.1 | 435.1988 | -0.2 | 3.90 | 17430 | +Na | 8 |
| 157 | 19β-Glucosyl-14- deoxyandrographoside | C_26_H_40_O_9_ | 496.2669 | 496.5669 | -0.3 | 519.2561 | -0.6 | 3.92 | 4472 | +Na | 48 |
| 158 | 25(S)-Ruscogenin | C_27_H_42_O_4_ | 430.3101 | 428.5101 | 1.8 | 453.2994 | 4.0 | 3.97 | 7940 | +Na | 36 |
| 159 | Trachelosperoside E-1 | C_36_H_58_O_12_ | 682.3947 | 680.5947 | 1.8 | 705.3839 | 2.6 | 3.98 | 12423 | +Na | 103 |
| 160 | Cimidahuside E | C_35_H_52_O_8_ | 600.3661 | 600.4661 | -0.1 | 601.3734 | -0.2 | 3.98 | 17896 | +H | 80 |
| 161 | Cimiaceroside A | C_35_H_54_O_9_ | 618.3770 | 618.177 | 0.2 | 619.3843 | 0.3 | 3.99 | 16748 | +H | 81 |
| 162 | Phytolaccagenic acid | C_31_H_48_O_6_ | 516.3460 | 515.346 | 1.0 | 539.3353 | 1.8 | 3.99 | 3240 | +Na | 38 |
| 163 | Esculentoside E | C_35_H_54_O_11_ | 650.3672 | 649.7672 | 0.6 | 673.3564 | 0.9 | 4.03 | 44783 | +Na | 83 |
| 164 | 25(S)-Ruscogenin | C_27_H_42_O_4_ | 430.3099 | 428.7099 | 1.6 | 453.2991 | 3.4 | 4.03 | 17113 | +Na | 32 |
| 165 | (Z)-(1S,5R)-β-Pinen-10- yl-β-vicianoside | C_21_H_34_O_10_ | 446.2151 | 446.3151 | -0.1 | 469.2043 | -0.2 | 4.04 | 4124 | +Na | 9 |
| 166 | 25(S)-Ruscogenin | C_27_H_42_O_4_ | 430.3099 | 428.7099 | 1.6 | 453.2991 | 3.4 | 4.04 | 16137 | +Na | 21 |
| 167 | Tussilagolactone | C_28_H_42_O_8_ | 506.2866 | 507.6866 | -1.4 | 507.2939 | -2.7 | 4.10 | 5997 | +H | 15 |
| 168 | Quinatoside D | C_39_H_60_O_11_ | 704.4151 | 702.9151 | 1.5 | 743.3782 | 2.1 | 4.11 | 15919 | +K | 66 |
| 169 | Melianol | C_35_H_48_O_9_ | 612.3298 | 612.3298 | 0.0 | 613.3371 | -0.1 | 4.12 | 111127 | +H | 23 |
| 170 | Semiaquilegoside A | C_26_H_38_O_9_ | 494.2503 | 495.5503 | -1.3 | 495.2576 | -2.6 | 4.15 | 2274 | +H | 16 |
| 171 | 11α,12α-Epoxy-3β,23- dihydroxy-30- norolean-20(29)- en-28,13β-olide | C_29_H_42_O_5_ | 470.3033 | 470.2033 | 0.1 | 471.3106 | 0.1 | 4.20 | 6724 | +H | 19 |
| 172 | Hookeroside C | C_38_H_62_O_15_ | 758.4080 | 759.308 | -0.9 | 759.4153 | -1.2 | 4.21 | 16821 | +H | 107 |
| 173 | Cimidahuside E | C_35_H_52_O_8_ | 600.3662 | 600.3662 | 0.0 | 601.3735 | 0.0 | 4.23 | 29970 | +H | 83 |
| 174 | Cimiaceroside A | C_35_H_54_O_9_ | 618.3771 | 617.9771 | 0.4 | 619.3844 | 0.6 | 4.24 | 8838 | +H | 54 |
| 175 | Cimiaceroside A | C_35_H_54_O_9_ | 618.3771 | 617.9771 | 0.4 | 619.3844 | 0.6 | 4.24 | 69987 | +H | 86 |
| 176 | 3-O- (β-D- Glucuronopyranosyl)- soyasapogenol B | C_36_H_58_O_9_ | 634.4087 | 633.8087 | 0.6 | 635.4160 | 0.9 | 4.25 | 9871 | +H | 67 |
| 177 | 3-O- (β-D- Glucuronopyranosyl)-  soyasapogenol B | C_36_H_58_O_9_ | 634.4062 | 636.3062 | -1.9 | 635.4134 | -3.0 | 4.26 | 2683 | +H | 63 |
| 178 | Picrasidine F | C_29_H_26_N_4_O_3_ | 478.2024 | 476.3024 | 1.9 | 479.2097 | 3.9 | 4.27 | 3925 | +H | 2 |
| 179 | Cimidahuside G | C_35_H_56_O_9_ | 620.3939 | 618.9939 | 1.4 | 621.4011 | 2.3 | 4.31 | 5453 | +H | 63 |
| 180 | Akebonoic acid | C_29_H_44_O_3_ | 440.3289 | 440.5289 | -0.2 | 441.3362 | -0.4 | 4.31 | 2527 | +H | 31 |
| 181 | 25- Anhydrocimigenol-3-O- β-D-xylopyranoside | C_35_H_54_O_8_ | 602.3816 | 602.5816 | -0.2 | 603.3889 | -0.4 | 4.32 | 8050 | +H | 54 |
| 182 | Hookeroside C | C_38_H_62_O_15_ | 758.4074 | 759.8074 | -1.4 | 759.4147 | -1.9 | 4.32 | 18419 | +H | 73 |
| 183 | Saikosaponin E | C_42_H_68_O_12_ | 764.4729 | 762.6729 | 1.8 | 803.4361 | 2.3 | 4.33 | 6602 | +K | 57 |
| 184 | Saikosaponin E | C_42_H_68_O_12_ | 764.4725 | 762.9725 | 1.5 | 803.4357 | 1.8 | 4.34 | 12310 | +K | 86 |
| 185 | 11α,12α-Epoxy-3β,23- dihydroxy-30- norolean-20(29)-  en-28,13β-olide | C_29_H_42_O_5_ | 470.3030 | 470.503 | -0.2 | 471.3103 | -0.4 | 4.35 | 14886 | +H | 29 |
| 186 | Cimicifugoside H2 | C_35_H_54_O_10_ | 634.3710 | 635.071 | -0.7 | 657.3603 | -1.0 | 4.36 | 18916 | +Na | 57 |
| 187 | Trachelosperoside D-1 | C_36_H_56_O_13_ | 696.3729 | 695.5729 | 0.8 | 719.3621 | 1.1 | 4.37 | 116769 | +Na | 92 |
| 188 | 30-Norhederagenin | C_29_H_44_O_4_ | 456.3245 | 455.8245 | 0.5 | 457.3318 | 1.2 | 4.39 | 8157 | +H | 63 |
| 189 | Platycogenic acid A lactone | C_36_H_54_O_12_ | 678.3615 | 678.4615 | -0.1 | 701.3507 | -0.1 | 4.43 | 10109 | +Na | 88 |
| 190 | Ganoderic acid H | C_32_H_44_O_9_ | 572.2978 | 573.0978 | -0.8 | 573.3050 | -1.4 | 4.43 | 3239 | +H | 20 |
| 191 | 19β-Glucosyl-14- deoxyandrographoside | C_26_H_40_O_9_ | 496.2688 | 494.6688 | 1.6 | 519.2580 | 3.1 | 4.48 | 7929 | +Na | 40 |
| 192 | 30-Norhederagenin | C_29_H_44_O_4_ | 456.3242 | 456.0242 | 0.3 | 457.3315 | 0.6 | 4.48 | 5239 | +H | 51 |
| 193 | Trachelosperoside D-1 | C_36_H_56_O_13_ | 696.3738 | 694.6738 | 1.7 | 719.3631 | 2.4 | 4.51 | 2963 | +Na | 15 |
| 194 | Trachelosperoside D-1 | C_36_H_56_O_13_ | 696.3738 | 694.6738 | 1.7 | 719.3631 | 2.4 | 4.51 | 76555 | +Na | 85 |
| 195 | 11α,12α-Epoxy-3β,23- dihydroxy-30- norolean-20(29)- en-28,13β-olide | C_29_H_42_O_5_ | 470.3035 | 470.1035 | 0.2 | 471.3107 | 0.5 | 4.51 | 13645 | +H | 28 |
| 196 | 11α,12α-Epoxy-3β,23- dihydroxy-30-  norolean-20(29)- en-28,13β-olide | C_29_H_42_O_5_ | 470.3024 | 471.1024 | -0.8 | 471.3097 | -1.6 | 4.52 | 24739 | +H | 46 |
| 197 | Azedarachin C | C_32_H_42_O_10_ | 586.2786 | 585.4786 | 0.8 | 587.2858 | 1.3 | 4.52 | 3333 | +H | 46 |
| 198 | 30-Norhederagenin | C_29_H_44_O_4_ | 456.3247 | 455.6247 | 0.7 | 457.3320 | 1.6 | 4.54 | 5311 | +H | 28 |
| 199 | 3β-Formyloxy-7β,12β- dihydroxy-4,4,14α- trimethyl-5α-  chol-11,15-dioxo-8-en-(E)-24-oic acid | C_28_H_40_O_8_ | 504.2738 | 502.7738 | 1.5 | 505.2811 | 2.9 | 4.56 | 12911 | +H | 11 |
| 200 | Hookeroside C | C_38_H_62_O_15_ | 758.4091 | 758.2091 | 0.2 | 781.3983 | 0.3 | 4.56 | 7924 | +Na | 80 |
| 201 | Cimidahuside E | C_35_H_52_O_8_ | 600.3667 | 599.8667 | 0.5 | 601.3740 | 0.8 | 4.57 | 38736 | +H | 63 |
| 202 | Cimidahuside E | C_35_H_52_O_8_ | 600.3666 | 600.0666 | 0.3 | 601.3738 | 0.6 | 4.57 | 17193 | +H | 42 |
| 203 | Cimiaceroside A | C_35_H_54_O_9_ | 618.3764 | 618.7764 | -0.4 | 619.3837 | -0.6 | 4.57 | 8082 | +H | 48 |
| 204 | Ganoderic acid MA | C_34_H_52_O_7_ | 572.3729 | 570.7729 | 1.6 | 573.3802 | 2.8 | 4.58 | 4712 | +H | 37 |
| 205 | Azedarachin C | C_32_H_42_O_10_ | 586.2797 | 584.3797 | 1.9 | 587.2870 | 3.2 | 4.62 | 4540 | +H | 66 |
| 206 | 25(S)-Ruscogenin | C_27_H_42_O_4_ | 430.3103 | 428.3103 | 2.0 | 453.2995 | 4.3 | 4.66 | 35880 | +Na | 31 |
| 207 | Cimidahuside E | C_35_H_52_O_8_ | 600.3666 | 599.9666 | 0.4 | 601.3739 | 0.6 | 4.69 | 56230 | +H, +Na | 65 |
| 208 | Ganoderic acid MA | C_34_H_52_O_7_ | 572.3717 | 572.0717 | 0.3 | 573.3789 | 0.6 | 4.69 | 3829 | +H | 34 |
| 209 | Morphine | C_17_H_19_NO_3_ | 285.1369 | 284.7369 | 0.4 | 286.1442 | 1.3 | 4.75 | 32102 | +H, +Na | 5 |
| 210 | Platycogenic acid A lactone | C_36_H_54_O_12_ | 678.3624 | 677.4624 | 0.9 | 701.3517 | 1.3 | 4.76 | 17904 | +Na | 98 |
| 211 | Cimidahuside E | C_35_H_52_O_8_ | 600.3670 | 599.567 | 0.8 | 601.3743 | 1.3 | 4.76 | 16229 | +H | 89 |
| 212 | Esculentoside O | C_35_H_54_O_10_ | 634.3701 | 635.9701 | -1.6 | 657.3593 | -2.5 | 4.79 | 22027 | +Na | 47 |
| 213 | 3-Hydroxy-12,14-  diene-27-nordeane-28- nic acid | C_29_H_42_O_4_ | 454.3087 | 453.9087 | 0.4 | 455.3160 | 0.9 | 4.79 | 8876 | +H | 50 |
| 214 | Esculentoside A | C_42_H_66_O_16_ | 826.4364 | 825.1364 | 1.3 | 849.4256 | 1.5 | 4.80 | 3644 | +Na | 121 |
| 215 | Phytolaccagenic acid | C_31_H_48_O_6_ | 516.3464 | 515.0464 | 1.3 | 539.3356 | 2.4 | 4.81 | 2106 | +Na | 24 |
| 216 | 11α,12α-Epoxy-3β,23- dihydroxy-30- norolean-20(29)-  en-28,13β-olide | C_29_H_42_O_5_ | 470.3034 | 470.1034 | 0.2 | 471.3107 | 0.4 | 4.82 | 10488 | +H | 25 |
| 217 | 11α,12α-Epoxy-3β,23- dihydroxy-30- norolean-20(29)-  en-28,13β-olide | C_29_H_42_O_5_ | 470.3036 | 470.0036 | 0.3 | 471.3108 | 0.7 | 4.82 | 5216 | +H | 14 |
| 218 | Prosapogenin 1 | C_38_H_58_O_12_ | 706.3922 | 707.0922 | -0.7 | 707.3994 | -0.9 | 4.83 | 13584 | +H | 62 |
| 219 | Cimidahuside J | C_37_H_56_O_11_ | 676.3839 | 674.7839 | 1.6 | 677.3911 | 2.4 | 4.86 | 2579 | +H | 40 |
| 220 | 19β-Glucosyl-14- deoxy-11,12-  didehydroand- rographoside | C_20_H_28_O_4_ | 332.1970 | 333.997 | -1.8 | 333.2043 | -5.3 | 4.89 | 27277 | +H | 5 |
| 221 | Pterodontoside H | C_21_H_36_O_7_ | 400.2462 | 400.1462 | 0.1 | 423.2355 | 0.3 | 4.90 | 2211 | +Na | 7 |
| 222 | Azedarachin C | C_32_H_42_O_10_ | 586.2781 | 585.9781 | 0.3 | 587.2854 | 0.6 | 4.98 | 40848 | +H | 25 |
| 223 | 3-Hydroxy-12,14- diene-27-nordeane-28- nic acid | C_29_H_42_O_4_ | 454.3092 | 453.4092 | 0.9 | 455.3165 | 2.1 | 4.98 | 2966 | +H | 34 |
| 224 | Azedarachin C | C_32_H_42_O_10_ | 586.2781 | 585.9781 | 0.3 | 587.2854 | 0.5 | 4.98 | 20104 | +H | 13 |
| 225 | Esculentoside A | C_42_H_66_O_16_ | 826.4369 | 824.6369 | 1.8 | 849.4261 | 2.2 | 5.02 | 6323 | +Na, +H | 127 |
| 226 | 14- Deoxyandrographolide | C_20_H_30_O_4_ | 334.2128 | 335.8128 | -1.6 | 335.2201 | -4.8 | 5.03 | 2668 | +H | 1 |
| 227 | Tomentogenin | C_21_H_36_O_5_ | 368.2569 | 367.5569 | 0.7 | 391.2461 | 1.7 | 5.07 | 6644 | +Na | 2 |
| 228 | 3β-For 3β-Formyloxy-7β,12β- dihydroxy-4,4,14α- trimethyl-5α-chol-11,15-dioxo-8-en-(E)-24-oic acid | C_28_H_40_O_8_ | 504.2734 | 503.1734 | 1.1 | 505.2807 | 2.2 | 5.08 | 3745 | +H | 16 |
| 229 | Tomentogenin | C_21_H_36_O_5_ | 368.2568 | 367.7568 | 0.5 | 391.2460 | 1.4 | 5.12 | 11781 | +Na | 0 |
| 230 | Thebaine | C_19_H_21_NO_3_ | 311.1524 | 310.9524 | 0.2 | 312.1596 | 0.7 | 5.14 | 12075 | +H, Na | 8 |
| 231 | Armepavine | C_19_H_23_NO_3_ | 313.1679 | 313.0679 | 0.1 | 314.1752 | 0.3 | 5.15 | 3225 | +H, Na | 4 |
| 232 | Phytolaccagenic acid | C_31_H_48_O_6_ | 516.3461 | 515.3461 | 1.0 | 539.3353 | 1.8 | 5.21 | 2641 | +Na | 9 |
| 233 | Thebaine | C_19_H_21_NO_3_ | 311.1525 | 310.8525 | 0.3 | 312.1598 | 1.1 | 5.25 | 4558 | +H, +Na | 5 |
| 234 | 26-Deoxyactein | C_37_H_56_O_10_ | 660.3886 | 659.0886 | 1.3 | 699.3518 | 1.9 | 5.28 | 9060 | +K | 35 |
| 235 | Chikusetsusaponin Ⅱ | C_42_H_66_O_14_ | 794.4435 | 796.2435 | -1.8 | 795.4507 | -2.3 | 5.29 | 13661 | +H | 29 |
| 236 | 23-Hydroxy-ursolic  acid-3-O-α-L-arabino- pyranosyl-(1-2)-β-D- glucuronopyranosyl-28- O-β-D-glucopyranoside | C_47_H_74_O_19_ | 942.4805 | 944.3805 | -1.9 | 943.4878 | -2.0 | 5.32 | 7732 | +H | 20 |
| 237 | Leonticine | C_20_H_25_NO_3_ | 327.1840 | 326.684 | 0.5 | 328.1913 | 1.6 | 5.34 | 3149 | +H, +Na | 3 |
| 238 | 25(R)-Ruscogenin-1-O- β-D-glucopyranosyl(1-2)-β-D- fucopyranoside | C_39_H_62_O_13_ | 738.4193 | 738.1193 | 0.3 | 761.4086 | 0.4 | 5.39 | 49058 | +Na | 25 |
| 239 | Chikusetsusaponin Ib | C_47_H_74_O_18_ | 926.4874 | 926.5874 | -0.1 | 927.4946 | -0.2 | 5.40 | 15044 | +H | 21 |
| 240 | 23-Hydroxy-ursolic acid-3-O-α-L-arabino- pyranosyl-(1-2)-β-D- glucuronopyranosyl-28- O-β-D-glucopyranoside | C_47_H_74_O_19_ | 942.4811 | 943.8811 | -1.4 | 943.4883 | -1.4 | 5.41 | 11154 | +H | 25 |
| 241 | Chikusetsusaponin Ib | C_47_H_74_O_18_ | 926.4864 | 927.5864 | -1.1 | 927.4937 | -1.2 | 5.49 | 40865 | +H | 34 |
| 242 | Cornutaside A | C_43_H_68_O_14_ | 808.4623 | 807.0623 | 1.4 | 809.4696 | 1.7 | 5.49 | 13592 | +H | 12 |
| 243 | Dauricoline | C_36_H_40_N_2_O_6_ | 596.2895 | 595.3895 | 0.9 | 597.2968 | 1.4 | 5.50 | 6039 | +H | 4 |
| 244 | Tomentogenin | C_21_H_36_O_5_ | 368.2564 | 368.1564 | 0.1 | 391.2456 | 0.3 | 5.50 | 3782 | +Na | 2 |
| 245 | Piperolactam-C9:1(8E) | C_20_H_27_NO_3_ | 329.1991 | 329.1991 | 0.0 | 330.2064 | 0.0 | 5.52 | 3404 | +H, Na | 0 |
| 246 | Kalmanol | C_20_H_34_O_6_ | 370.2337 | 372.1337 | -1.9 | 371.2410 | -5.0 | 5.53 | 2513 | +H | 1 |
| 247 | Chikusetsusaponin Ⅳ (Araloside A, PJS-3) | C_47_H_74_O_18_ | 926.4858 | 928.1858 | -1.7 | 927.4931 | -1.8 | 5.55 | 19448 | +H | 20 |
| 248 | Trifoside A | C_46_H_72_O_18_ | 912.4732 | 911.0732 | 1.4 | 913.4805 | 1.5 | 5.58 | 4083 | +H | 15 |
| 249 | Vitetrifolin E | C_22_H_36_O_4_ | 364.2610 | 364.561 | -0.3 | 365.2683 | -0.9 | 5.67 | 3184 | +H, +Na | 11 |
| 250 | Dauricoline | C_36_H_40_N_2_O_6_ | 596.2892 | 595.7892 | 0.5 | 619.2784 | 0.9 | 5.70 | 17529 | +Na,  +H | 4 |
| 251 | 25(R)-Ruscogenin-1-O- β-D-glucopyranosyl (1-2)-β-D- fucopyranoside | C_39_H_62_O_13_ | 738.4207 | 736.7207 | 1.7 | 761.4100 | 2.2 | 5.70 | 3803 | +Na | 23 |
| 252 | Pipernonaline | C_21_H_27_NO_3_ | 341.1993 | 340.9993 | 0.2 | 342.2066 | 0.7 | 5.71 | 11491 | +H,  +Na | 4 |
| 253 | Tetrandrine | C_38_H_42_N_2_O_6_ | 622.3051 | 621.5051 | 0.8 | 623.3124 | 1.3 | 5.76 | 4003 | +H, +Na | 7 |
| 254 | Vitetrifolin E | C_22_H_36_O_4_ | 364.2617 | 363.9617 | 0.3 | 365.2690 | 0.9 | 5.76 | 19914 | +H, +Na | 6 |
| 255 | Mubenoside A | C_45_H_72_O_17_ | 884.4775 | 883.9775 | 0.5 | 907.4667 | 0.6 | 5.78 | 18465 | +Na | 14 |
| 256 | Dehydrosoyasaponin | C_48_H_76_O_18_ | 940.5023 | 941.3023 | -0.8 | 941.5096 | -0.9 | 5.84 | 2748 | +H | 28 |
| 257 | Dauricoline | C_36_H_40_N_2_O_6_ | 596.2889 | 596.0889 | 0.2 | 619.2781 | 0.4 | 5.84 | 2283 | +Na | 1 |
| 258 | Dauricoline | C_36_H_40_N_2_O_6_ | 596.2888 | 596.0888 | 0.2 | 619.2780 | 0.3 | 5.84 | 33197 | +Na, +H | 8 |
| 259 | Prosapogenin 2 | C_32_H_48_O_8_ | 560.3336 | 561.7336 | -1.4 | 561.3408 | -2.4 | 5.84 | 2527 | +H, +K | 14 |
| 260 | Vitetrifolin E | C_22_H_36_O_4_ | 364.2612 | 364.3612 | -0.1 | 365.2685 | -0.3 | 5.86 | 13419 | +H | 7 |
| 261 | Piperolein B | C_21_H_29_NO_3_ | 343.2149 | 343.1149 | 0.1 | 344.2221 | 0.4 | 5.87 | 66749 | +H, +Na | 3 |
| 262 | Piperolein B | C_21_H_29_NO_3_ | 343.2148 | 343.1148 | 0.1 | 366.2040 | 0.2 | 5.88 | 2651 | +Na, +H | 2 |
| 263 | Dauricoline | C_36_H_40_N_2_O_6_ | 596.2889 | 595.9889 | 0.3 | 619.2782 | 0.5 | 5.92 | 2828 | +Na | 1 |
| 264 | Hordatine B | C_29_H_40_N_8_O_5_ | 580.3131 | 579.3131 | 1.0 | 619.2763 | 1.5 | 5.93 | 3574 | +K | 9 |
| 265 | Clinopodiside C | C_48_H_76_O_18_ | 940.5040 | 939.704 | 0.8 | 941.5113 | 0.9 | 5.93 | 6137 | +H | 19 |
| 266 | Dauricoline | C_36_H_40_N_2_O_6_ | 596.2889 | 595.9889 | 0.3 | 619.2782 | 0.5 | 5.93 | 69515 | +Na, +H | 13 |
| 267 | Dauricoline | C_36_H_40_N_2_O_6_ | 596.2889 | 595.9889 | 0.3 | 619.2781 | 0.5 | 5.93 | 2055 | +Na | 1 |
| 268 | Tetrandrine | C_38_H_42_N_2_O_6_ | 622.3058 | 620.8058 | 1.5 | 623.3131 | 2.4 | 5.93 | 2826 | +H | 2 |
| 269 | Mubenoside A | C_45_H_72_O_17_ | 884.4774 | 884.0774 | 0.4 | 907.4666 | 0.4 | 5.93 | 32261 | +Na | 17 |
| 270 | Quinatoside B | C_40_H_62_O_13_ | 750.4184 | 751.1184 | -0.7 | 751.4257 | -0.9 | 5.98 | 5945 | +H | 13 |
| 271 | Mubenoside A | C_45_H_72_O_17_ | 884.4765 | 884.8765 | -0.4 | 907.4658 | -0.5 | 5.99 | 3200 | +Na | 19 |
| 272 | Mubenoside A | C_45_H_72_O_17_ | 884.4781 | 883.3781 | 1.1 | 907.4673 | 1.3 | 5.99 | 48130 | +Na | 18 |
| 273 | Rubiprasin A | C_32_H_52_O_5_ | 516.3822 | 515.6822 | 0.7 | 517.3894 | 1.3 | 6.07 | 2779 | +H | 3 |
| 274 | Tetrandrine | C_38_H_42_N_2_O_6_ | 622.3044 | 622.2044 | 0.1 | 645.2936 | 0.2 | 6.08 | 24813 | +Na, +H | 9 |
| 275 | Prosapogenin 1 | C_38_H_58_O_12_ | 706.3917 | 707.5917 | -1.2 | 707.3989 | -1.7 | 6.09 | 11137 | +H | 21 |
| 276 | Platycodigenin | C_30_H_48_O_7_ | 520.3410 | 519.341 | 1.0 | 543.3302 | 1.8 | 6.10 | 5479 | +Na | 3 |
| 277 | Dauricoline | C_36_H_40_N_2_O_6_ | 596.2894 | 595.4894 | 0.8 | 619.2786 | 1.3 | 6.14 | 18578 | +Na, +H | 4 |
| 278 | Ophiopogonin C' | C_39_H_62_O_12_ | 722.4247 | 721.8247 | 0.6 | 745.4139 | 0.8 | 6.20 | 29686 | +Na | 13 |
| 279 | Hyptadienic acid | C_30_H_46_O_4_ | 470.3392 | 470.7392 | -0.4 | 471.3465 | -0.8 | 6.20 | 2624 | +H | 3 |
| 280 | Tetrandrine | C_38_H_42_N_2_O_6_ | 622.3053 | 621.3053 | 1.0 | 645.2945 | 1.6 | 6.21 | 19654 | +Na, +H | 5 |
| 281 | Belamcandal | C_32_H_48_O_6_ | 528.3437 | 529.7437 | -1.4 | 529.3510 | -2.6 | 6.23 | 2737 | +H | 12 |
| 282 | Guineensine | C_24_H_33_NO_3_ | 383.2461 | 383.2461 | 0.0 | 406.2353 | 0.1 | 6.28 | 7920 | +Na, +H | 0 |
| 283 | Guineensine | C_24_H_33_NO_3_ | 383.2464 | 382.9464 | 0.3 | 384.2536 | 0.8 | 6.28 | 80342 | +H, +Na | 15 |
| 284 | Tetrandrine | C_38_H_42_N_2_O_6_ | 622.3049 | 621.7049 | 0.6 | 645.2941 | 1.0 | 6.33 | 36543 | +Na, +H | 5 |
| 285 | Arborane | C_30_H_52_ | 412.4054 | 413.9054 | -1.5 | 435.3946 | -3.4 | 6.36 | 9987 | +Na | 0 |
| 286 | Saikosaponin D | C_42_H_68_O_13_ | 780.4647 | 781.7647 | -1.3 | 781.4719 | -1.7 | 6.42 | 19827 | +H | 12 |
| 287 | Saponin PA | C_44_H_70_O_16_ | 854.4653 | 855.5653 | -1.1 | 855.4726 | -1.3 | 6.43 | 2872 | +H | 10 |
| 288 | Hederagenin-3-O-β-D- xylopyranosyl-(1 2)-α-  L-arabinopyranoside | C_40_H_64_O_12_ | 736.4381 | 738.1381 | -1.7 | 737.4454 | -2.3 | 6.45 | 24771 | +H | 16 |
| 289 | Lucidenic acid N  (Lucidenic acid LM1) | C_27_H_40_O_6_ | 460.2823 | 460.4823 | -0.2 | 461.2896 | -0.4 | 6.46 | 2100 | +H | 1 |
| 290 | Arjunolic acid | C_30_H_48_O_5_ | 488.3513 | 487.2513 | 1.1 | 489.3585 | 2.2 | 6.52 | 5787 | +H | 5 |
| 291 | 2β,3β,19α- Trihydroxyurs-12-en-28- oic acid | C_30_H_48_O_5_ | 488.3513 | 487.2513 | 1.1 | 489.3586 | 2.2 | 6.53 | 2857 | +H | 3 |
| 292 | Ginsenoside F5 | C_41_H_70_O_13_ | 770.4824 | 769.7824 | 0.7 | 793.4716 | 0.9 | 6.53 | 10774 | +Na | 17 |
| 293 | Yesanchinoside C | C_47_H_80_O_19_ | 948.5289 | 949.0289 | -0.5 | 949.5362 | -0.5 | 6.58 | 6172 | +H | 54 |
| 294 | Mubenoside A | C_45_H_72_O_17_ | 884.4750 | 886.475 | -2.0 | 885.4822 | -2.2 | 6.58 | 6996 | +H | 67 |
| 295 | Mubenoside A | C_45_H_72_O_17_ | 884.4759 | 885.5759 | -1.1 | 885.4832 | -1.2 | 6.58 | 131532 | +H | 69 |
| 296 | Ginsenoside F5 | C_41_H_70_O_13_ | 770.4832 | 768.8832 | 1.6 | 793.4724 | 2.0 | 6.61 | 12607 | +Na | 59 |
| 297 | (25S)-26-O-β-D- Glucopyranosyl-5β-  furost-20(22)- en-3β,15,26-triol-3-O-[α-L-rhamnopyranosyl  (1 4)]-β-D- glucopyranoside | C_46_H_78_O_18_ | 918.5177 | 919.6177 | -1.1 | 919.5250 | -1.2 | 6.61 | 6659 | +H | 19 |
| 298 | 3-O-α-L- Rhamnopyranosyl-(1-2)-α-L-  arabinopyranosylgypso genin | C_41_H_64_O_12_ | 748.4378 | 750.4378 | -2.0 | 749.4451 | -2.6 | 6.62 | 17368 | +H | 74 |
| 299 | Dehydroabietic acid | C_20_H_28_O_2_ | 300.2072 | 302.0072 | -1.8 | 323.1964 | -5.5 | 6.62 | 3678 | +Na | 0 |
| 300 | 4,8,12-Trimethyl- tridecanoic acid | C_16_H_32_O_2_ | 256.2422 | 254.2422 | 2.0 | 279.2314 | 7.0 | 6.62 | 3169 | +Na | 0 |
| 301 | 25- Anhydrocimigenol-3-O- β-D-xylopyranoside | C_35_H_54_O_8_ | 602.3807 | 603.5807 | -1.2 | 603.3879 | -2.0 | 6.62 | 9357 | +H | 30 |
| 302 | Azedarachin C | C_32_H_42_O_10_ | 586.2797 | 584.3797 | 1.9 | 609.2689 | 3.2 | 6.63 | 3155 | +Na | 14 |
| 303 | Soyasaponin βg | C_47_H_74_O_17_ | 910.4940 | 909.094 | 1.4 | 911.5013 | 1.5 | 6.64 | 7078 | +H | 39 |
| 304 | Vitetrifolin E | C_22_H_36_O_4_ | 364.2616 | 364.0616 | 0.20 | 387.2508 | 0.5 | 6.66 | 72518 | +Na, +H | 2 |
| 305 | Vitetrifolin E | C_22_H_36_O_4_ | 364.2613 | 364.2613 | 0.0 | 387.2505 | -0.1 | 6.71 | 2529 | +Na | 6 |
| 306 | Vitetrifolin E | C_22_H_36_O_4_ | 364.2613 | 364.2613 | 0.0 | 387.2505 | -0.1 | 6.72 | 37060 | +Na, +H | 11 |
| 307 | Notoginsenoside R1 | C_47_H_80_O_18_ | 932.5330 | 933.933 | -1.4 | 933.5403 | -1.5 | 6.74 | 28146 | +H | 19 |
| 308 | 23-Hydroxy-betulinic acid | C_30_H_48_O_4_ | 472.3562 | 471.3562 | 1.0 | 473.3635 | 2.0 | 6.79 | 3614 | +H | 3 |
| 309 | Luteoxanthin | C_40_H_56_O_4_ | 600.4194 | 598.8194 | 1.6 | 623.4087 | 2.5 | 6.82 | 3139 | +Na | 3 |
| 310 | Oleanolic acid 3-O-β-D- xylopyranosyl-(1-2)-α- L-arabinopyranoside | C_40_H_64_O_11_ | 720.4458 | 719.5458 | 0.90 | 743.4350 | 1.3 | 6.83 | 38995 | +Na | 24 |
| 311 | Akebonoic acid | C_29_H_44_O_3_ | 440.3279 | 441.5279 | -1.2 | 463.3171 | -2.5 | 6.83 | 2141 | +Na | 2 |
| 312 | Raddeanoside R0 | C_35_H_56_O_7_ | 588.4041 | 586.9041 | 1.5 | 611.3933 | 2.5 | 6.87 | 6337 | +Na | 4 |
| 313 | Paeonenolide F | C_32_H_46_O_5_ | 510.3344 | 510.4344 | -0.1 | 511.3417 | -0.3 | 6.89 | 2298 | +H | 2 |
| 314 | Chuanbeinone | C_27_H_43_NO_2_ | 413.3275 | 415.1275 | -1.8 | 414.3348 | -4.4 | 6.90 | 2271 | +H | 1 |
| 315 | 1α-Angeloyloxy-7β-(4- methylsenecioyloxy) oplopa-3(14)Z,8(10)- dien-2-one | C_26_H_36_O_5_ | 428.2547 | 429.7547 | -1.5 | 429.2620 | -3.6 | 6.94 | 2704 | +H | 0 |
| 316 | Olibanumols H | C_30_H_52_O_3_ | 460.3917 | 460.3917 | 0.0 | 483.3809 | 0.1 | 6.95 | 4636 | +Na | 2 |
| 317 | 3β-Hydroxyurs-12- en-27,28-dioic acid | C_30_H_46_O_5_ | 486.3334 | 487.4334 | -1.1 | 509.3226 | -2.3 | 6.96 | 2090 | +Na | 17 |
| 318 | Ophiopogonin C' | C_39_H_62_O_12_ | 722.4228 | 723.7228 | -1.3 | 723.4301 | -1.8 | 7.02 | 66619 | +H | 30 |
| 319 | 19α-Hydroxy-3-acetyl- ursolic acid | C_32_H_50_O_5_ | 514.3643 | 515.8643 | -1.5 | 515.3716 | -2.9 | 7.04 | 5919 | +H | 9 |
| 320 | Methyl glycyrrhetate | C_31_H_48_O_4_ | 484.3543 | 485.3543 | -1.0 | 485.3616 | -2.0 | 7.06 | 2813 | +H | 6 |
| 321 | Kalopanaxsaponin I | C_46_H_74_O_16_ | 882.4971 | 883.0971 | -0.6 | 883.5044 | -0.6 | 7.10 | 5296 | +H | 16 |
| 322 | Astragaloside | C_47_H_76_O_17_ | 912.5079 | 912.9079 | -0.4 | 913.5152 | -0.4 | 7.19 | 8354 | +H | 6 |
