# Supplementary material for: Dataset on LC-Q-TOF/MS tentative identification of phytochemicals in the extract of Vernonia amygdalina leaf through positive ionization
Source: Data Brief. 2018 Nov 3;21:1686–9. doi: 10.1016/j.dib.2018.10.159 (PMC6249520; doi:10.1016/j.dib.2018.10.159)
Supplement: Supplementary file 1 — Supplementary material [file mmc1.docx]

Alara, Oluwaseun Ruth,

Faculty of Chemical Engineering & Natural Resources Engineering

Universiti Malaysia Pahang, Tun Razak, 26300, Gambang, Pahang,

Malaysia

ruthoalao@gmail.com

25th July, 2018.

Editor-in-Chief

Data in Brief,

Dear editor,

This manuscript describes an original article and is not under consideration by any other journal. All authors approved the manuscript and submission for your consideration in Data in Brief. Please find the enclosed manuscript entitled “Dataset on **LC-Q-TOF/MS tentative identification of phytochemicals in the extract of *Vernonia amygdalina* leaf through positive ionization**” by ^*^Oluwaseun Ruth Alara, Nour Hamid Abdurahman, Chinonso Ishmael Ukaegbu, Zulkafli Hassan, and Nassereldeen Ahmed Kabbashi

We believe that this manuscript is appropriate for publication by Data in Brief because it presents the data on identification of secondary metabolites in the plant extract.

We have no conflicts of interest to disclose. If you feel that the manuscript is appropriate for your journal, we suggest the following reviewers:

1. Martin G. Schmid

Department for Pharmaceutical Chemistry, Institute for Pharmaceutical Sciences, University of Graz, Graz, Austria

e-mail: [martin.schmid@uni-graz.at](mailto:martin.schmid@uni-graz.at)

2. Olugbenga Solomon Bello

Department of Pure and Applied Chemistry, Ladoke Akintola University of Technology Ogbomoso, Nigeria

e-mail: osbello06@gmail.com; [osbello@lautech.edu.ng](mailto:osbello@lautech.edu.ng)

Tel: +2348035685435

3. Chandrasekar Uma

Associate Professor, Biochemistry, Hawasaa University, Ethiopia

e-mail: umachandrasekaran29@gmail.com

We hope that the enclosed manuscript and reviewer suggestions fulfil the requirements for publication in Data in Brief. Thank you for receiving our manuscript and considering it for review. We appreciate your time and look forward to your response.

Yours Sincerely,

Alara, Oluwaseun Ruth Phd (in view)

Faculty of Chemical and Natural Resources Engineering,

Universiti Malaysia Pahang
